# Complex resistivity characteristics of saltwater-intruded sand contaminated by heavy metal

**DOI:** 10.1038/s41598-019-47167-8

**Published:** 2019-07-29

**Authors:** Changxin Nai, Xiaochen Sun, Zeya Wang, Ya Xu, Yuqiang Liu, Jingcai Liu, Lu Dong, Qifei Huang, Yuling Wang

**Affiliations:** 1 School of Information and Electronic Engineering, Shandong Technology and Business University, Yantai, 264005 China; 2 School of Computer Science and Technology, Shandong Technology and Business University, Yantai, 264005 China; 3 State Key Laboratory of Environmental Criteria and Risk Assessment, Chinese Research Academy of Environmental Sciences, Beijing, 100012 China; 4 Department of information and electrical engineering, Shandong Jianzhu University, Jinan, 250101 China

**Keywords:** Chemical physics, Geochemistry, Chemical physics, Geochemistry

## Abstract

Different pollutants affect electrical characteristics of soil, e.g., electric resistivity and capacity. The most extensively used non-intrusive methods in mapping these physical characteristics are electrical method. To better understand the effect of different hydrogeological and environmental process on resistivity and phase of complex resistivity under water-saturated soil, we carried out a controlled laboratory experiment where the host material was simulated by sand soil and the hydrogeological and environmental processes by groundwater table rise, seawater intrusion and heavy metal contamination. The experiment measured the resistivity and phase of soil saturated and unsaturated, with different pollutants added, together with their time-lapse change in a well-controlled column. With the involvement of more measurement parameters, complex resistivity method can provide more information than resistivity method, thereby having better performance in the detection and monitoring of changes in electrical properties of complex contaminated sites. For example, it is capable of discriminating the different contamination process, in this case, e.g., seawater intrusion and heavy metal contamination. In addition, it is still sensitive to the change of pollutant concentration even in site with high added concentration. Furthermore, simulating the saltwater-intruded site contaminated by manganese, it was found that the change of resistivity (ρ) can hardly be observed, while the responses of phase (φ) are so obvious that can be clearly observed.

## Introduction

In recent years, geophysical techniques in the detection and monitoring of contaminants such as heavy metals and persistent organic pollutants (POPs) have been extensively employed in the restoration of contaminated soil and groundwater^1–3^. A large number of studies have demonstrated the validity of geophysical methods in the detection and mapping of the soil or groundwater contaminated by those pollutants^4,5^. The information gained from non-intrusive geophysical methods, in conjunction with water sampling, shows the potential in exploring the contamination extent, with time and cost savings in mind^6,7^. The significance of a detailed characterization and precise mapping of the source and distribution of contamination for a remediation project has been increasingly acknowledged by scientific community^8,9^.

However, the potential of geophysical data to assess contaminated sites has not been fully utilized so far; for example, area with low resistivity can be mapped by high-density electrical resistivity tomography (ERT) but it is difficult to find connection between the resistivity to the specific soil conditions^10,11^. This is due to the resistivity characteristics of soil can change due to certain conditions such as: intrinsic porosity of grains and sediments, content of aqueous fluid and air, besides those related to the chemical variations of groundwater^12^, and to the insufficiency of studies devoted to finding detailed answers to such questions.

The limitation of ERT on discriminating different contaminants is more prominent when it is applied to investigate the contaminated sites with multiple pollutants, such as combined effect of seawater intrusion and heavy metal pollution. This is because groundwater table in coastal areas is generally high, which leads to a high moisture content, and consequently low background resistivity of the soil^13^. Under such circumstance, the low resistivity caused by the heavy metal pollution may be obscured and difficult to be delineated. Due to the seawater intrusion, coastal soils often contain more ions, such as Na^+^, Cl^−^ etc., which makes it difficult to distinguish whether the low resistance is the result of heavy metal pollution or seawater intrusion^14^.

The complex resistivity(CR) method may solve this problem well^15^. The CR method is a geophysical technique that has originally been used for ore prospecting, but currently also for hydrogeological and environmental purposes^16,17^. Compared to the traditional ERT method, it can provide more information (phase and imaginary part of complex resistivity, etc.) to identify the electrical differences caused by different factors (moisture content, contamination type or concentration)^18^. For example, Olhoeft^19^ observed the difference in phase response between uncontaminated and organic-contaminated clay soil, which stimulated further research on interaction of clay between different organic pollutants and associated changes in CR signature. Furthermore, CR responses to different phases of DNAPL(Dense Non-Aqueous Phase Liquid) within groundwater aquifer were also studied^20^. However, for the CR response to sites with ions, or with multiple pollutants, there are only a few studies. Most of the studies are still in the laboratory stage, and few researches have been conducted on field scale^21,22^.

The aim of this study is to develop a better understanding of the sensitivity of CR method to the different pollutants and not to analyse the complex chemical and electrochemical process that occur in the experimental setting over time. The study is focused on pointing out the effectiveness of CR method in distinguishing the difference of electrical properties caused by pollutant type and concentration. This work was motivated by the fact that, if the different conductive ions exhibits different complex resistivity signatures under water-saturated soil, opportunities may exist for non-invasive site characterization of heavy metal contaminated site in those coastal areas high in groundwater table and rich in conductive ions due to the intrusion of seawater.

## Materials and Methods

### Sample preparation and experimental treatments

**Figure 1:**
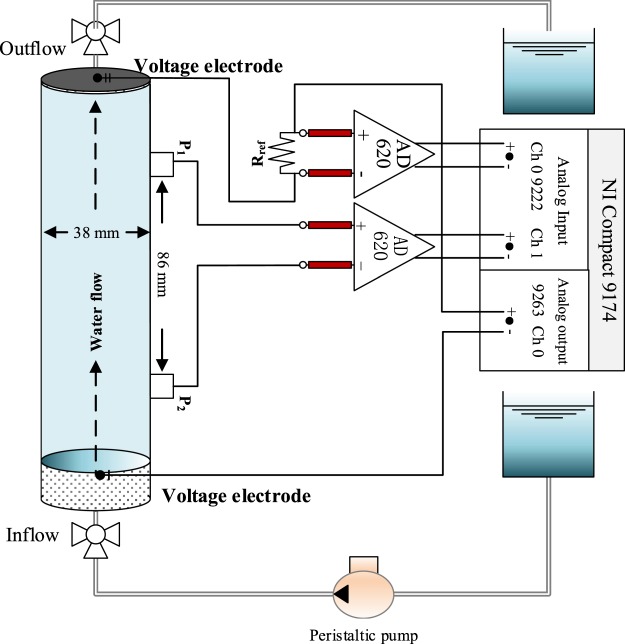
Schematic of experimental setup showing the sample holder and the main components for the CR data acquisition system.

We constructed sandy soil contaminated with heavy metal similar to those used in Personna^15^. Metallic-free soils were created from a mixture of Ottawa sand (SiO_2_ > 99%, specific gravity = 2.65, and d_50_ = 0.5 × 10^−3^ m) and clay (bentonite). The sand (95% w/w) and clay (5% w/w) were mechanically mixed in an effort to render the mixture as homogenous as possible in order to avoid the presence of preferential flow. To create the experimental samples, soils were filled into cylindrical transparent PMMA columns with a diameter of 3.8 cm and a length of 30 cm (Fig. 1). A 0.45 μm PVDF membrane was placed on the bottom and top of each column to prevent the sand from migrating out of the column under convection. Each sample was constructed in triplicate. The triplicate sample construction was chosen in an effort to assess repeatability and quantify changes in CR spectra between samples that could result simply from the sample packing procedure irrespective of changes in pollutant concentration.

In order to simulate pollution and further encourage complete saturation, all liquids flow through the bottom to the top of the sample. The saturating fluid was either water, NaCl or MnCl_2_ solution, which were made from pure NaCl or MnCl_2_ and groundwater(23.63 × 10^−3^ S/m at 26.0 °C, major anions: Cl^−^, and SO_4_^2-^ and minor anions: F^−^, and NO_3_^−^; major cations: Na^+^, K^+^, Ca^2+^, and Mg^2+^ and minor cations: Cu^2+^, Mn^2+^, Zn^2+^, Fe^2+^/Fe^3+^, and Al^3+^). All measurements were performed at room temperature (24–26 °C). The CR response of the various samples was monitored for up to 7 days or longer until the data were stable.

**Figure 2:**
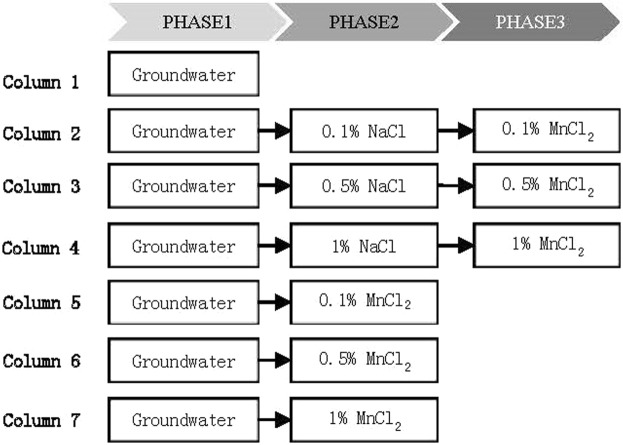
Experiments to simulate different processes of hydrology and pollution.

The whole experiment process was divided into three experimental stages, hereafter referred as Phase I, Phase II and Phase III (Fig. 2) based on the experimental treatments applied for complex resistivity measurements. Experiment columns were divided into three groups, and different treatments were applied to them. The column 1 in the first group was saturated with water to simulate the CR characteristics after groundwater table rise. The column 2~4 in the second group were saturated with NaCl solution to examine the CR characteristic of coastal soil after seawater intrusion; and the other three columns were saturated with MnCl_2_ solution to examine the CR characteristics of Mn-contaminated site. In Phase III, MnCl_2_ solution was added to the column 2~4 to reflect the properties of seawater invaded sites contaminated by heavy metals.

### CR instrumentation

We performed CR measurements (2^−4^, 2^−3^, 2^−2^, 2^−1^, 1, 2, 2^2^, 2^3^, 2^4^ and 2^5^ Hz) using a dynamic signal analyser (NI 9174) with respect to a reference resistance (Rref). A preamplifier (AD620) was used to ensure a high input impedance, thereby reducing instrumentation errors. The CR measurements were recorded using a four-electrode configuration: two Copper spiral electrodes placed at the ends of the column were used to inject current into the sample and two Ag-AgCl electrodes were used to record potential difference signals (Fig. 1).

**Figure 3:**
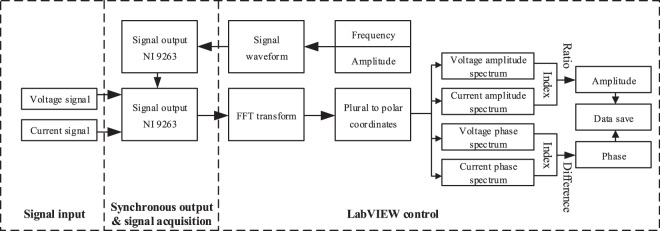
Framework of CR data acquisition system.

The whole process of data collection and measurement (Fig. 3) was as follows: first, through the LabVIEW program, sinusoidal AC signals with different frequency were outputted by controlling the signal output module NI 9263, while the voltage and current signal of the sample were obtained by controlling the data acquisition module NI 9222. Then performing the Fast Fourier Transform(FFT) to obtain the spectral data of the voltage and current and indexing the amplitude and phase at the desired frequency to calculate ratio and difference. Finally, we calculated the amplitude and phase of CR and saved data to facilitate the latter comparative analysis or inversion imaging.

### Calibration measurements

**Figure 4:**
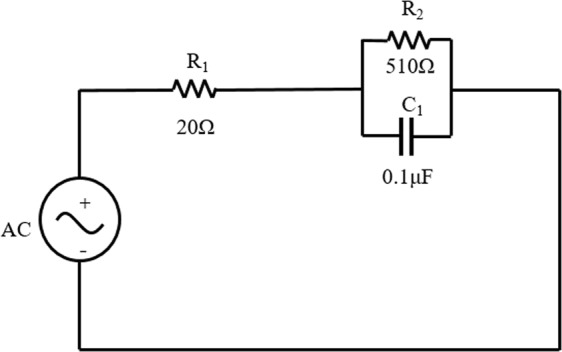
Analogous circuit schematic.

**Figure 5:**
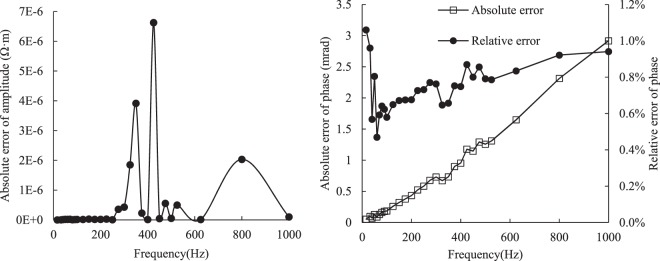
Measurement error of complex resistivity in analogous circuit.

In order to verify the validity and accuracy of CR measurement system, a measurement circuit was designed in which AC was the power supply signal, R_1_ was the current reference resistor and R_2_ was the end of CR acquisition (Fig. 4). Because the parameters of each unit are known, the amplitude and phase can be calculated, and then the measurement error can be obtained by comparing with the measured data (Fig. 5).

It can be seen in the figure that there is almost no error in the amplitude and the measurement error of the phase is almost less than 1%. Therefore, we considered that the accuracy of measurement system is guaranteed.

## Result and Discussion

### CR response to different contamination levels

**Figure 6:**
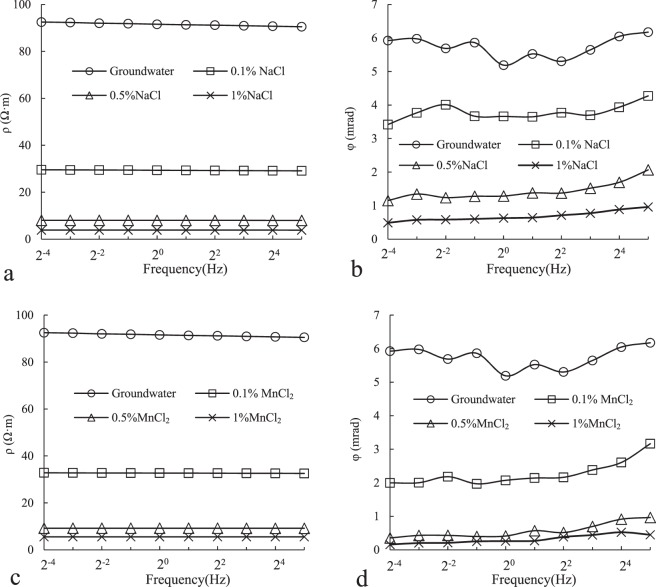
Simulation of CR characteristics of soil under different pollutant concentration conditions.

Figure 6 shows the measurement results of CR with same contaminants and different contamination levels. From Fig. 6 we can see that both φ(phase) and ρ(resistivity) decrease obviously as salinity increased. For example, when the concentration of NaCl raised from 0 to 0.1%, 0.5% and 1%,ρ (the maximum value under different frequency conditions) decreased from 92.51 to 29.58, 8.05, 3.86, φ (the maximum value under different frequency conditions) decreased from 6.17 to 4.27, 2.06, and 0.96 too.

However, although both φ and ρ decrease with salinity, there are subtle differences between them. ρ changed obviously (2.7 times), even slightly larger than φ (2.2 times) at lower concentration (from 0.1% to 0.5%). While a little change with ρ(1.07 times) and a big change with φ (1.33 times) at higher concentration (from 0.5% to 1%). This is consistent with other research, such as Yoon^23^ found that with the fixed moisture content and other parameters, the resistivity had a large change even at low concentrations of leachate. As the concentration increased, the resistivity was no longer sensitive to the change of concentration. This phenomenon becomes more pronounced in saturated soils.

We know that the change of phase is closely related to dielectric properties of materials, which is mainly caused by the polarization of various kinds of charge carriers. When an external electric field is applied, the charge distribution realign in materials. In porous materials, two polarization mechanisms are generally accepted over the frequency range 1 mHz~1 kHz: (1) Electric double layer (EDL, <100 Hz) polarization and (2) Maxwell-Wagner (MW, ≥100 Hz) polarization. As the theory of MW polarization is fairly well-known, the research on EDL polarization has increased significantly in recent years. If an alternating electric field is applied, there are three polarization mechanisms exist on the grain-electrolyte interface: Stern layer polarization, diffuse layer polarization and membrane polarization^24,25^. Previous studies have suggested that diffuse layer polarization is likely strongly attenuated in porous materials with contiguity between the grains because of the overlap of the electrical diffuse layer. They thought this polarization occurs at frequencies much higher than the low-frequency range used in the present investigation^26,27^. Leroy *et al*.^28^ and Schmutz *et al*.^29^ thought the membrane polarization contribution can be neglected in the frequency range 1 Hz to 1 MHz. Therefore, the electrochemical polarization may be due to the polarization of the Stern layer alone. Because the charge exchange rate between the Stern layer and the diffuse layer is too slow with respect to the characteristic time scale of the pulsation of the electric field, it can be considered that there is no ion exchange between the Stern layer and the diffuse layer. The cations of the Stern layer move tangentially along the layer and accumulate on one side of the grains in the electric field direction.

The results of Leroy *et al*.^27^ showed that the phase decreases with salinities, Revil *et al*.^22^ discussed salinity dependence of spectral induced polarization in sands and also got the same result. Although salinity dependence was observed, it cannot be explained by existing theories because they neglected the role of diffuse layer polarization. In this paper, we mainly discussed orientation polarization of water molecules and diffuse layer polarization which have been neglected.

**Figure 7:**
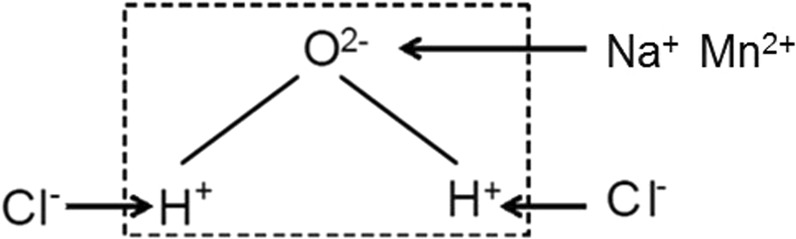
Interaction of anions and cations in solution with water molecules (>100 Hz).

If there is only water in the column, the orientation of water molecules is almost unrestricted because the content of free ion is low, so a stronger orientation polarization and a larger phase, this mechanism was often overlooked. When the solution contains NaCl or MnCl_2_, ions interact with water molecules, cations (Na^+^, Mn^2+^) are attracted by the oxygen to the negative end of the water dipole and Cl^−^ is attracted to the hydrogen of the water molecules, which leads to a lower orientation polarization (Fig. 7)^30,31^. The higher salt concentration in solution, the more inhibition of water dipole rotation, and the lower effect of orientation polarization.

**Figure 8:**
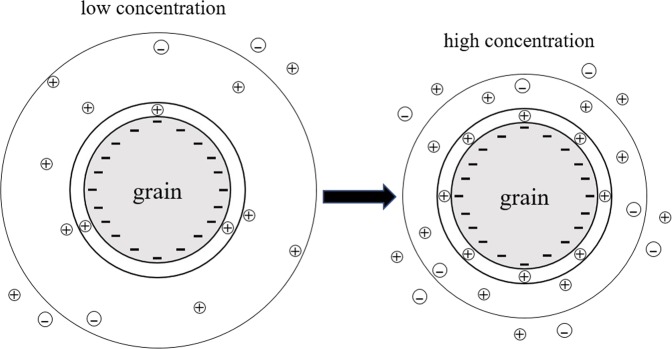
Deformation of electrical double layer on grain surface with concentration changes.

The mechanisms responsible for generating a CR signature in soils are not yet fully understood. Within the range of frequencies studied in this paper, the polarization response is dominated by the electrical double layer as shown by Leroy *et al*.^27^ and Vaudelet *et al*.^26^. However, we focued on the diffuse layer polarization and considered that the diffuse layer polarization can’t be neglected. At the interface between soil and water, when salt concentration changes, the change of free ion content in the solution leads to the electrical double layer change^32^. Although the concentration of coions with the same electrical properties as grains is high with the increase of salt concentration in solution, all these ions(anions) are excluded from the interface because of electrostatic repulsion. The increase of counterions(cations) will largely enter Stern layer, which will neutralize negative charge on the surface of grains and reduce the net charge (Fig. 8). So the electrostatic attraction of the cations in the diffuse layer is reduced, and the thickness of diffuse layer decreases, zeta potential decreases, finally leading to inhibition of the polarization on electrical double layer. It is noted that when the solution concentration is high enough, the cations in Stern layer completely neutralized the negative charge on the surface of grain, and then there is no diffuse layer.

Based on this phenomenon, when the pollution is less, the resistivity is sensitive to the change of pollution concentration, so resistivity method can better define the contamination levels. But as the concentration increases such as in a high concentration pollution source zone, the sensitivity of resistivity to the change of concentration decreases. However, when the pollution is heavy such as in the pollution source zone, the phase is more meaningful because it is more sensitive to the salinity.

### CR responses varied with different geochemical process

**Figure 9:**
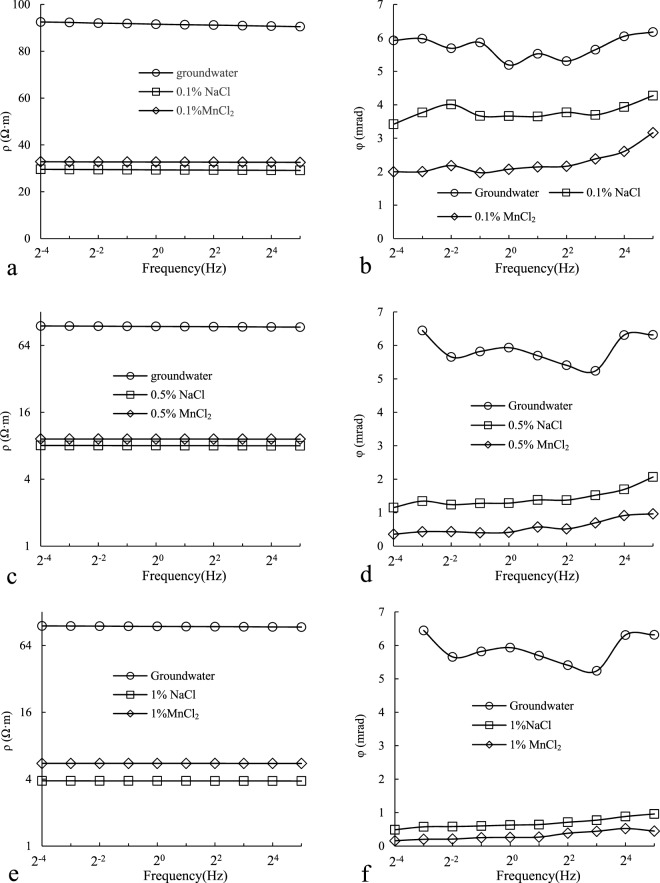
Simulation of CR characteristics of soil under different hydrological processes and pollution conditions.

Figure 9 shows the CR characteristics of soil under different soil conditions. Figure 9 suggests that there is a significant difference in resistivity between water saturated soil column and salt solution saturated soil column. For example, there was a 3-fold difference in resistivity between 0.1%NaCl saturated soil column and water saturated soil column. While the concentration of NaCl was increased to 0.5%, difference increased for resistivity by a factor of 11.5. The difference between MnCl_2_ saturated soil column and water saturated soil column was relatively small, but still up to 2.8 times (MnCl_2_ 0.1%) and 10.0 times (0.5%), respectively. The difference between NaCl and MnCl_2_ saturated soil column was only 10% (0.1%) and 12% (0.5%) under the same concentration. The difference in resistivity under the same concentration and different pollutant type is very small, so it is difficult to distinguish.

From the comparison of φ in Fig. 9, the relative difference between water saturated soil column and saturated soil column of NaCl and MnCl_2_ is smaller than that of ρ. For example, the maximum difference in φ between 0.1%NaCl saturated soil column and water saturated soil column appeared at 2^−4^Hz, and it’s only 0.7 times. Although the concentration increased to 0.5%, the difference has increased, but only 5.1 times, far less than the difference in ρ at the same concentration. This difference suggests that resistivity is better than phase response to indicate groundwater table rise as opposed to seawater intrusion or heavy metal pollution.

Comparing NaCl saturated soil column and MnCl_2_ saturated soil column, we found a obvious difference in φ with the same concentration of pollutants but not in ρ. For example, when salt concentration was 0.1%, the ratio of ρ between NaCl and MnCl_2_ was about 1: 1.1 under different conditions. Although the ratio of φ was different when frequency changed (the highest was 1:1.93 at 2^−2^ Hz, the lowest was 1:1.35 at 2^5^ Hz), it was significantly larger than that of ρ. As the concentrations were increased to 0.5%, the phase difference was more obvious. The highest was 1:3.12 (2^−3^ Hz) and the lowest was 1:1.86 (2^4^ Hz). Obviously, compared to the resistivity, CR phase is better to distinguish different contaminants especially heavy pollution situation. It should be pointed out that the phase difference of different contaminants is more obvious with the increase of concentration, and the value of phase is futher reduced.

Kaya *et al*.^30^ compared the difference of dielectric response caused by NaCl and CaCl_2_ pollution at the same concentration, results showed that the inhibition of polarization by CaCl_2_ is stronger than that by NaCl, which is similar to the results in this paper. This is related to the ionic strength of pore fluid. The higher ionic strength of CaCl_2_ or MnCl_2_ decreases the thickness of the diffuse double layer more than that of NaCl at the same concentration. This is also the theoretical basis for CR method to distinguish two cations.

### CR response of soil contaminated by heavy metals after seawater intrusion

**Figure 10:**
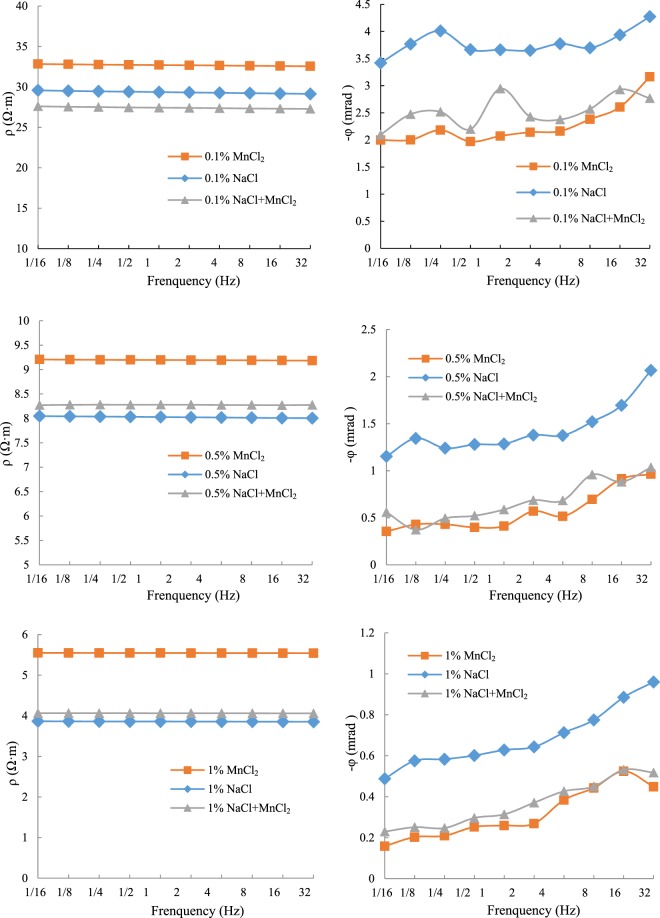
Simulation of CR characteristics of soil after being intruded by seawater and polluted by heavy metals.

Studies have shown that affected by tides, the recharge, runoff and discharge conditions of groundwater are dynamically changed in coastal areas: seawater recharge groundwater at high tide and groundwater recharge seawater at low tide. This leads to higher Na^+^, K^+^, Cl^−^ and lower background resistivity in the soil and groundwater of coastal areas. In this case, whether the electrical abnormality caused by heavy metal pollution can be detected, and whether the pollution degree can be accurately distinguished will greatly affect the application of electrical method in coastal contaminated sites. Therefore, we added MnCl_2_ solution to soil column which has been contaminated by NaCl solutions and observed the change of electrical properties (Fig. 10).

It can be seen from Fig. 10 that ρ changed little, but φ changed greatly after adding the same concentration of MnCl_2_ solution in NaCl saturated soil column. Taking 0.1%NaCl soil column as an example (2^−1^Hz), ρ was about 27.5 Ω·m after adding 0.1%MnCl_2_ which was originally about 29.4 Ω·m, a decrease of 6.5%. But φ decreased from 3.67 mrad to 2.20 mrad, a decrease of 40.1%. A similar phenomenon was observed in other NaCl saturated soil columns: ρ changed relatively little and φ changed relatively greatly after adding MnCl_2_. This revealed that in groundwater - seawater intersections or the areas affected by seawater intrusion, it was difficult to detect heavy metal pollution through traditional resistivity method.

Further analysis shows that the resistivity of NaCl saturated soil column is not changed after adding MnCl_2_ solution, which is very close to the resistivity of using NaCl alone. However the phase changes are relatively large and it is very close to the phase of using MnCl_2_ alone. Therefore, after adding MnCl_2_, the polarization effect of Mn^2+^ suppresses Na^+^ and dominants the polarization process because manganese is a divalent cation, it can forms relatively strong (less mobile) complexation with both inner and outer sphere bondings more so than sodium^26^.

## Conclusions

To better understand the effect of different hydrogeological and environmental process on resistivity and phase of complex resistivity under water-saturated soil, we carried out a controlled laboratory experiment where the host material was simulated by soil and the hydrogeological and environmental processes by groundwater table rise, seawater intrusion and heavy metal contamination. The experiment measured the resistivity and phase of soil saturated and unsaturated, with different pollutants, together with their time-lapse change in a well-controlled column. Several conclusions have been made as follow by analyzing the experiment result:

The two process of water table rising and contamination induce different responses both in φ and ρ, which indicates that both resistivity method and CR method are capable of monitoring and discriminating the two different processes. Different pollutants with the same concentration induced almost the same responses of soil resistivity. However change of φ they induced are significantly different, which indicates the potential of CR of discriminating the different contamination process, in this case, e.g., seawater intrusion and manganese contamination. For the same pollutant, the φ seems to be more sensitive than ρ to the change of concentration as the concentration is large to some extent, which means CR’s potential of characterizing heavily contaminated areas (e.g. source zone) in detail. After simulating the combined effect of seawater intrusion and heavy metal pollution, it was found that the relative change of ρ can hardly be observed, while the responses of φ are so obvious that can be clearly observed. In conclusion, the combining of resistivity and CR method makes it possible to characterize contaminant distribution and behavior of a complex well-characterized site with multiple pollutants.
